# Enhancement of presynaptic glutamate release and persistent inflammatory pain by increasing neuronal cAMP in the anterior cingulate cortex

**DOI:** 10.1186/1744-8069-4-40

**Published:** 2008-09-29

**Authors:** Long-Jun Wu, Hendrik W Steenland, Susan S Kim, Carolina Isiegas, Ted Abel, Bong-Kiun Kaang, Min Zhuo

**Affiliations:** 1 Department of Physiology, Faculty of Medicine, University of Toronto Centre for the Study of Pain, University of Toronto, 1 King's College Circle, Toronto, Ontario M5S 1A8, Canada; 2 Department of Biology, University of Pennsylvania, Philadelphia, PA 19104, USA; 3 National Creative Research Initiative Center for Memory, Department of Biological Sciences, Seoul National University, San 56-1 Silim-dong Gwanak-gu, Seoul 151-747, Korea

## Abstract

Both presynaptic and postsynaptic alterations are associated with plastic changes of brain circuits, such as learning and memory, drug addiction and chronic pain. However, the dissection of the relative contributions of pre- and postsynaptic components to brain functions is difficult. We have previously shown peripheral inflammation caused both presynaptic and postsynaptic changes and calcium-stimulated cyclic AMP (cAMP) pathway in the anterior cingulate cortex (ACC) is critical in the synaptic plasticity and behavioral sensitization to pain. It remains to be elucidated whether presynaptic or postsynaptic modulation by cAMP in the ACC could be sufficient for enhancing inflammatory pain. In order to address this question, we took advantage of a novel transgenic mouse model, heterologously expressing an *Aplysia *octopamine receptor (Ap oa_1_). This receptor is G protein-coupled and selectively activates the cAMP pathway. We found that activation of Ap oa_1 _by octopamine enhanced glutamatergic synaptic transmission in the ACC by increasing presynaptic glutamate release *in vitro*. Bilateral microinjection of octopamine into the ACC significantly facilitated behavioral responses to inflammatory pain but not acute pain. The present study provides the first evidence linking enhanced presynaptic glutamate release in the ACC to behavioral sensitization caused by peripheral inflammation.

## Background

Central synaptic plasticity, including long-term potentiation (LTP) and long-term depression (LTD), is thought to be a cellular basis for multiple brain functions, such as learning and memory, drug addition, and persistent pain [1-3]. Both enhancement of presynaptic glutamate release and increased postsynaptic glutamate receptor-mediated responses have been reported to contribute to LTP of excitatory synapses and plasticity-related behavioral consequences. For example, conditional fear memory is reported to trigger LTP in lateral amygdala [4], where both recruitment of AMPA receptor and enhanced glutamate release are involved [5,6]. In ventral tegmental area – nucleus accumbens pathway, drug of abuse induced postsynaptic and/or presynaptic plasticities [7,8]. Similarly, in the anterior cingulate cortex (ACC), a brain area related to persistent pain, peripheral inflammation caused functional alterations in postsynaptic NMDA receptors and presynaptic glutamate release [9,10]. However, the dissection of the relative contributions of pre- and postsynaptic components to these plasticity-related brain functions is difficult. Although presynaptic enhancement of glutamate release is associated with these plastic changes in brain function, there is no study available to directly address the physiological or pathological significance of presynaptic plasticity.

Among many possible candidate molecules, cyclic AMP (cAMP) is a key second messenger for synaptic plasticity and different forms of memory across different species, from invertebrates to vertebrates, including *Aplysia*, *Drosophila*, mice and rats [11-13]. Presynaptically, cAMP and cAMP-dependent protein kinase A (PKA) could target to presynaptic ion channels, such as potassium channels and the hyperpolarization-activated cation channel, or to presynaptic exocytosis machinery [14-16]. Postsynaptically, cAMP-PKA pathway is involved in AMPA receptor trafficking and activation of gene transcription that required for the late-phase of LTP [1,17,18]. Behaviorally, the role of cAMP in learning and memory also has been well documented. Genetic mutants lacking adenylyl cyclases (ACs), PKA, or cAMP response element binding protein (CREB) exhibit memory defects [11,19-21].

It has been proposed that chronic pain and long-term memory share some common synaptic mechanisms [22-24]. Consistent with this notion, plasticity in sensory synapses located at pain-processing brain regions was shown to contribute to chronic pain [25,26]. For example, in the ACC, cAMP pathway was demonstrated to be critical for synaptic plasticity and behavioral sensitization to pain. We found impaired LTP in the ACC [27] and attenuated behavioral sensitization in various chronic pain models in mice lacking calmodulin-stimulated AC1 and AC8 [28,29]. Moreover, both postsynaptic and presynaptic alterations in the ACC after chronic pain are shown to be mediated by cAMP pathway [9,10,30]. However, genetic approaches and pharmacological approaches did not address the contribution of presynaptic *vs *postsynaptic mechanisms in the cortex to behavioral persistent pain. It is unclear if changes in the ACC, or even changes in presynaptic glutamate release may be sufficient to cause behavioral nociceptive responses.

While genetic and pharmacological approaches have been useful in examining the role of the cAMP pathway in pain, a combination of these approaches provides unique chance for us to investigate synaptic mechanisms related to inflammatory pain by using transgenic mice with heterologously expressed receptors impacting the cAMP pathway. We took advantage of transgenic mice heterologously expressing an *Aplysia *octopamine receptor (Ap oa_1_) [31]. This G protein coupled receptor selectively activates the cAMP pathway after binding of its natural ligand, octopamine [32], in forebrain. Our results show that activation of Ap oa_1 _in the ACC enhanced glutamatergic synaptic transmission by increasing presynaptic glutamate release *in vitro *and enhanced responses to inflammatory pain *in vivo *by bilateral microinjection of octopamine. The current study provides direct evidence that increased presynaptic glutamate release by cAMP pathway in the ACC is sufficient for chronic pain.

## Methods

### Animals

Both WT mice and Ap oa_1 _transgenic mice were described in a recent report [31]. Experiments were performed in mice aged between 8–12 weeks old. All mice were maintained on a 12 h light/dark cycle with food and water provided *ad libitum*. The Animal Studies Committee at the University of Toronto approved all experimental protocols.

### Brain slice preparation

Adult male WT or transgenic mice (8–12 weeks old) were anesthetized with 1–2% halothane. Coronal brain slices (300 μm) containing the ACC were prepared using standard methods [33]. Slices were transferred to a submerged recovery chamber with oxygenated (95% O_2 _and 5% CO_2_) artificial cerebrospinal fluid (ACSF) containing (in mM): 124 NaCl, 2.5 KCl, 2 CaCl_2_, 2 MgSO_4_, 25 NaHCO_3_, 1 NaH_2_PO_4_, 10 glucose at room temperature for at least 1 h.

### Whole-cell patch clamp recordings in adult ACC slices

After one hr recovery, slices were placed in a recording chamber on the stage of an Olympus BX51WI microscope (Tokyo, Japan) with infrared DIC optics for visualization of whole-cell patch clamp recordings. Excitatory postsynaptic currents were recorded from layer II/III neurons with an Axon 200B amplifier (Molecular Devices, CA) in ACC and stimulations were delivered by a bipolar tungsten stimulating electrode placed in layer V of the ACC. Recording electrodes (2–5 MΩ) contained a pipette solution composed of (in mM): K-gluconate, 120; NaCl, 5; MgCl_2 _1; EGTA, 0.5; Mg-ATP, 2; Na_3_GTP, 0.1; HEPES, 10; pH 7.2; 280–300 mOsmol. To examine the voltage dependence of EPSCs, Cs-MeSO_3 _was used to replace the K-gluconate. To record miniature EPSCs (mEPSCs), TTX (1 μM) was added in the bath solution. Access resistance was 15–30 MΩ and was monitored throughout the experiment. In cases of experiments related to comparison between control and injected mice, stimulation intensity was adjusted to produce similar magnitude of EPSC responses. Data were discarded if access resistance changed more than 15% during an experiment. The membrane potential was held at -70 mV throughout the experiment. When recording NMDA EPSCs, a holding potential of -30 mV was used as indicated.

Drugs were applied to the perfusion solution. All drugs were purchased from Sigma Aldrich. In some experiments, a picopump (WPI pneumatic picopump, Sarasota, FL) was used for local application of glutamate [34]. Before establishing whole-cell recording, the drug application pipette was moved beside the neuron using a micromanipulator (Sutter MP-285, Novato, CA). The tip of the pipette was about 5–10 μm away from the neuron recorded. The diameter of the drug application pipette tip was about 3–4 μm. The pressure and duration of the puff was 15 psi and 100 ms, respectively.

### Brain Cannulation surgery

Mice were anesthetized with isoflurane (1–3%, as needed) inhalation with 30% oxygen balanced with Nitrogen. The scalp was shaved and then cleaned with iodine (Triadine) and alcohol. The head of the mouse was fixed into a stereotaxic adapter (GENEQ Inc. Model 463013, Montreal, Quebec City, CA) mounted on a stereotaxic frame (Kopf Model 962, Tujunga, CA, USA) and lubricant (Artificial Tears) was applied to the eyes. An incision was made over the skull and the surface exposed. Two small holes were drilled above the ACC and the dura was gently reflected. Guide cannula were placed so that the final coordinates of the microinjection would be 0.7 mm anterior to Bregma, 0.3 mm lateral to the midline, and 1.75 mm ventral to the surface of the skull [28].

### Microinjection of Octopamine and behavioral tests

Mice were restrained in a plastic cone (Braintree Scientific) and a small whole was cut in the plastic overlying the microinjection guides. The dummy cannula was removed and the microinjection cannula was inserted into the guide. For microinjection, a 30 gauge injection cannula was used which was 0.8 mm lower than the guide. Microinjection was conducted using a motorized syringe pump (Razel Scientific Instruments Inc., Stamford, Connecticut) and a Hamilton syringe. Octopamine (1 mM), dissolved in saline, was delivered to left and right ACC (500 nL in 1 minute) through the cannula. The volume delivered was confirmed by watching the movement of the meniscus down a length of calibrated polyethylene (PE10) tubing. Following delivery to each side of the brain, the injection cannula was left in place for 1 minute to help prevent any solution from flowing back up the guide. The cannula was then retracted and inserted into the opposite side of the brain.

After the last microinjection, formalin (5%, 10 μl) was injected subcutaneously into the dorsal side of the left hindpaw. The total time between the first microinjection and the formalin injection was at most 6 minutes. Following hind paw injection, the animals were immediately delivered to a clear plastic cylinder for behavioral observation. The total time spent licking or biting the injected hindpaw was recorded for each 5 min interval over the course of 2 hr. Upon completion of experiments, animals were deeply anesthetized and perfused transcardially with saline, followed by 4% paraformaldehyde. Brains were dehydrated overnight in a 30% sucrose solution for cryoprotection. Brains were serially sectioned on a cryostat at (30 μm) and were mounted on glass slides. The sections were then stained with Hematoxylin-Eosin (H&E) and observed under microscope to confirm the site of injection.

For acute pain tests, mice were gently restrained in a plastic cone for bilateral microinjection of 1 mM octopamine (as described above). 1 minute after the last microinjection the tail-flick reflex of the mouse was tested. The spinal tail-flick reflex was evoked by focused radiant heat applied to the underside of the tail. A photocell timer measured the latency to reflexive removal of the tail away from the heat. Following the tail-flick reflex test the animal was removed from the plastic cone and placed in its home cage for 5 minutes. The animals were then tested on the hotplate test. For this test, mice were placed on a thermally controlled metal plate (Columbia Instruments, Columbus, Ohio). The time between the placement of a mouse on the plate and licking or lifting of a hind paw was measured with a digital timer. The temperature of the hotplate was set to 55.0°C. Mice were removed from the hot plate immediately from the first response.

### Data analysis and statistics

Miniature EPSCs were detected and analyzed using an event detection program (Mini Analysis Program; Synaptosoft, Inc., Decatur, GA). The threshold for detecting mEPSCs was set as 1.5 time of the noise level. Results were analyzed by t-test, paired t-test, or two-way ANOVA followed by post-hoc Student-Newman-Keuls test to identify significant differences. All data are expressed as mean ± S.E.M. In all cases, *P *< 0.05 was considered statistically significant.

## Results

We have previously shown that inflammatory pain caused synaptic alterations in the ACC. For example, presynaptic glutamate release is enhanced while postsynaptic NMDA NR2B subunit is upregulated in the ACC pyramidal neurons after peripheral inflammation [9,10]. In addition, forebrain overexpression of NR2B is sufficient for enhancement of inflammatory pain [35]. However, there is no study on the regional and temporal manipulations in synaptic transmission in the ACC and chronic pain. Octopamine is a trace amine and its endogenous level is far below those of the classical neurotransmitters [36]. Using transgenic mice heterologously expressing Ap oa_1_, we could selectively activate cAMP pathway in the ACC and examine its effect in the synaptic transmission and behavior sensitization. The same mice were also reported to exhibit enhanced hippocampal synaptic plasticity and fear memory [31].

### Normal neuronal excitability and glutamatergic excitatory neurotransmissions in the ACC pyramidal neurons in transgenic mice

**Figure 1:**
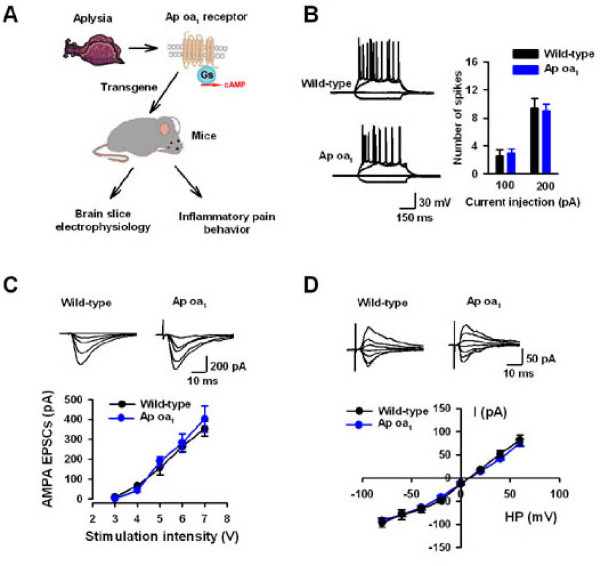
**No difference in neuronal excitability or excitatory neurotransmissions in ACC pyramidal neurons from WT and Ap oa_1 _mice**. (*A*) Diagram illustrating the experimental design and procedures. Ap oa_1 _receptor is a Gs-coupled receptor from *Aplysia*. Transgenic mice expressing Ap oa_1 _receptors were used for slice electrophysiology and inflammatory pain behaviors. (*B*) Representative traces and pooled results showing neuronal responses to current injections from -200 pA to 200 pA with 100 pA step for 400 ms. Action potentials were induced in neurons from both WT and transgenic mice. (*C*) Sample trances and pooled results showing no difference in input-output curve of AMPA receptor-mediated EPSCs between WT and Ap oa_1 _mice. (*D*) No difference in I-V curve of AMPA receptor-mediated EPSCs between WT and transgenic mice.

To examine whether heterologous expression of Ap oa_1 _affects neuronal and synaptic properties of ACC neurons, we compared the neuronal excitability and basal synaptic transmissions in wild-type (WT) and transgenic mice. Conventional whole-cell patch clamp recordings were performed in pyramidal neurons from layer II/III of the ACC. Pyramidal neurons in the ACC were selected by their morphology and spike properties [33]. We found that there was no difference in the resting membrane potential of ACC pyramidal neurons between WT (-65.1 ± 2.1 mV, n = 25 neurons/15 mice) and Ap oa_1 _mice (-64.3 ± 1.8 mV, n = 22 neurons/8 mice). No difference was found in the number of spikes in neurons from wild-type and transgenic mice with current injections of either 100 pA or 200 pA (n = 22 neurons/8–12 mice for each group) (Figure 1A and 1B).

Glutamatergic transmissions in the ACC were then examined in Ap oa_1 _mice. AMPA receptor-mediated excitatory postsynaptic currents (EPSCs) were recorded in layer II/III pyramidal neurons by stimulating layer V in the ACC. In the presence of GABA_A _antagonist, picrotoxin (100 μM) and NMDA receptor antagonist, AP5 (50 μM), AMPA EPSCs were isolated. The input-output relationship of AMPA receptor-mediated EPSCs was examined. Different stimulation intensities were applied and the amplitude of EPSCs was compared between WT and transgenic mice. We found there was no difference in the input-output curve of AMPA EPSCs in between two groups (Figure 1C). To examine the voltage dependence of AMPA EPSCs, we recorded the current over a range of membrane potentials from -85 mV to +55 mV. No difference was found in the current-voltage (I-V) curve of AMPA EPSCs in WT and transgenic mice (Figure 1D).

**Figure 2:**
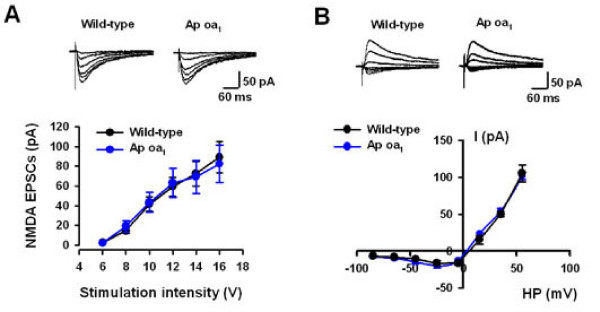
**Normal NMDA receptor-mediated EPSCs in Ap oa_1 _mice**. (*A*)No difference in input-output curve of NMDA receptor-mediated EPSCs between WT and Ap oa_1 _mice. (*B*) No difference in I-V curve of NMDA receptor-mediated EPSCs between WT and Ap oa_1 _mice.

NMDA receptor in the ACC is critical for synaptic plasticity and chronic pain [9,35,37]. Therefore, we want to know whether the NMDA receptor-mediated EPSCs were normal in the transgenic mice. NMDA EPSCs were isolated in the presence of picrotoxin (100 μM) and non-NMDA receptor antagonist, CNQX (20 μM). Similarly, the input-output relationship and I-V curve were compared between two groups. We found that there was no significant difference in either input-output or I-V curve in ACC neurons from WT and transgenic mice (Figure 2A and 2B).

### Octopamine enhanced glutamatergic transmission in transgenic mice

**Figure 3:**
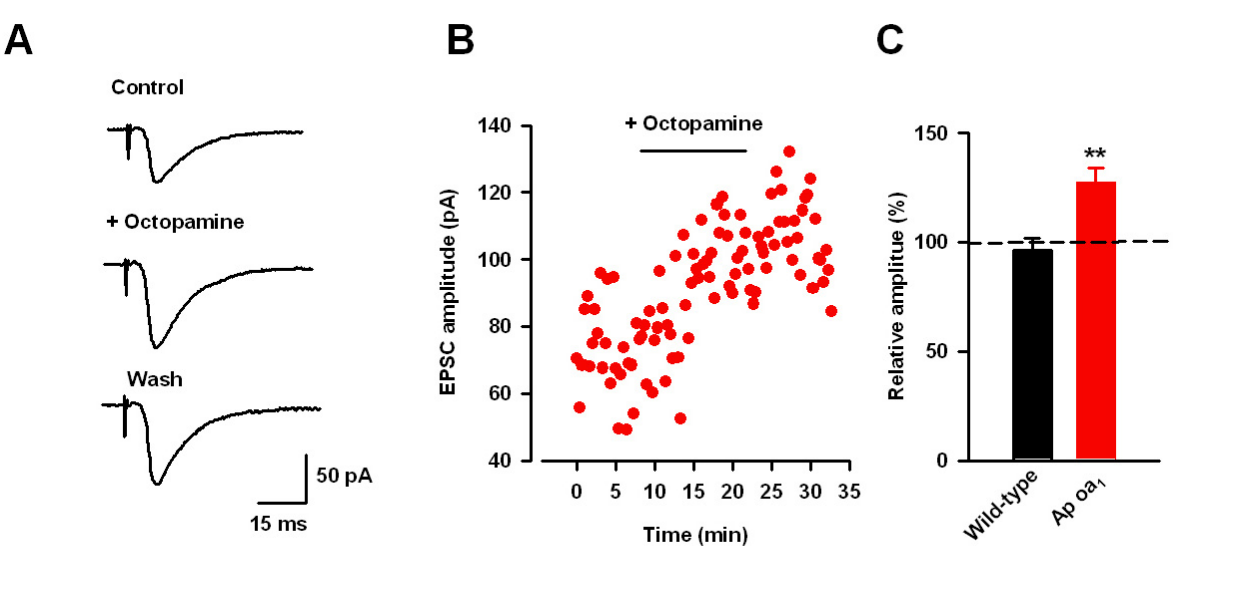
**Enhancement of EPSCs by octopamine in transgenic but not WT mice**. (*A*) Sample traces showing EPSCs before and after application of octopamine (50 μM) in Ap oa_1 _mice. (*B*) The time course of octopamine's effect on EPSCs in a neuron shown in A. (*C*) Pooled data showing octopamine significantly increased EPSCs in transgenic but not WT mice.

It is well known that Ap oa_1 _is selectively coupled to Gs protein [38] and activation of heterologous expression of Ap oa_1 _in HEK293 cells selectively stimulated cAMP synthesis after octopamine application [32]. Next, we wanted to test the function of exogenous Ap oa_1 _in ACC pyramidal neurons in transgenic mice. AMPA EPSCs were isolated and octopamine (50 μM) was then bath applied after stable EPSCs were obtained. In most neurons tested (8 of 11 neurons/8 mice), octopamine significantly increased the amplitude of EPSCs 10 minutes after the drug application (127.5 ± 6.8% of control, n = 11, P < 0.01, Figure 3A–C). The enhanced EPSCs by octopamine is long lasting, only showing partial recovery after 10 minutes washout of octopamine. In the WT mice, however, octopamine had no significant effect on the amplitude of EPSCs (96.5 ± 5.2% of control, n = 5, P = 0.57, Figure 3C). Therefore, octopamine exerts their stimulatory effect on glutamatergic neurotransmission via octopamine receptors in the transgenic mice.

**Figure 4:**
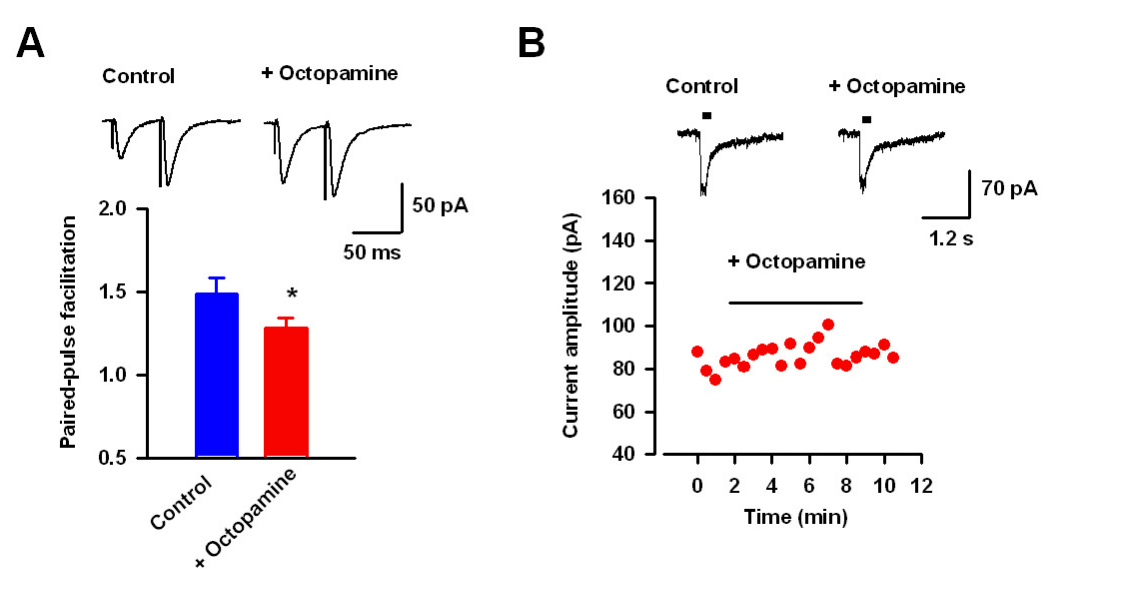
**Presynaptic action of octopamine in transgenic mice**. (*A*) Representative traces (upper) and pooled results (lower) showing that octopamine significantly decreased pair-pulse facilitation in transgenic mice. (*B*) Octopamine (50 μM) had no effect on puff-applied glutamate-induced current in transgenic mice.

The enhanced amplitude of EPSCs by octopamine in Ap oa_1 _mice may be due to increased function of postsynaptic AMPA receptor or presynaptic glutamate release. To address the issue, we tested pair-pulse ratio, a commonly used criteria to study the presynaptic release [10], in ACC pyramidal neurons by octopamine in transgenic mice. Paired stimuli with 50 ms intervals were applied and pair-pulse facilitation (PPF) was calculated before and after application of octopamine in transgenic mice (Figure 4A). We found that bath application of octopamine (50 μM) significantly decreased PPF from 1.48 ± 0.09 to 1.28 ± 0.06 (n = 14, P < 0.05, paired t-test, Figure 4A). Therefore, the results demonstrate the enhanced presynaptic glutamate release after activation of Ap oa_1 _by application of octopamine.

### Octopamine had no effect on the glutamate receptor-mediated currents in transgenic mice

The enhanced amplitude of EPSCs and decreased PPF suggests that octopamine may increase the presynaptic glutamate release but not the function of postsynaptic AMPA receptor. To confirm the idea, glutamate was locally applied by puff to induce glutamate receptor-mediated currents and then octopamine was bath-applied in transgenic mice. Glutamate (100 μM) induced inward currents in ACC pyramidal neurons, which may be mainly mediated by AMPA receptors (Figure 4B). Glutamate was applied every 30 s and bath application of octopamine (50 μM) for 10 minutes has no effect on the glutamate receptor-mediated currents (95.9 ± 4.8% of control, n = 5, P = 0.91, Figure 4B). These results confirm that activation of octopamine receptor by octopamine has no effect on the channel function of glutamate receptors.

### Octopamine increase mEPSC frequency but not amplitude in transgenic mice

**Figure 5:**
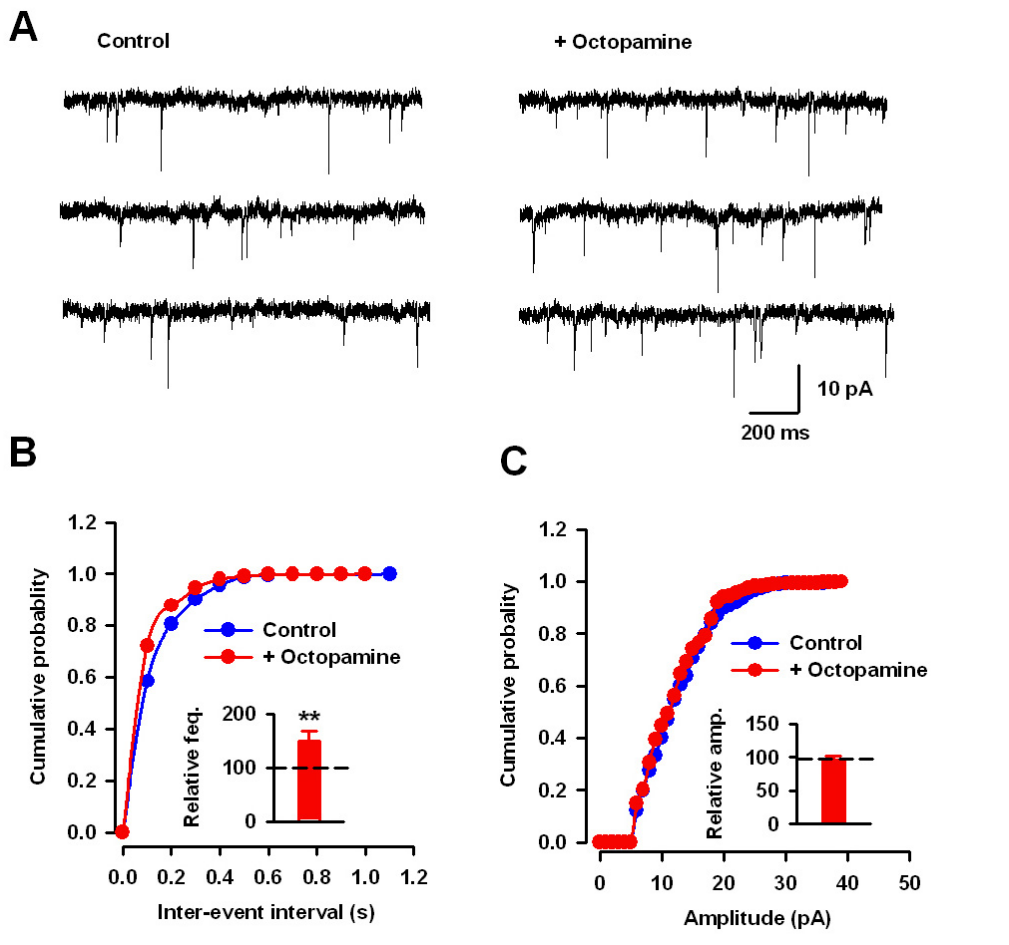
**Enhancement of mEPSC frequency by octopamine in transgenic mice**. (*A*) Sample traces showing the effect of octopamine (50 μM) on mEPSCs in Ap oa_1 _mice. (*B*) The cumulative probability plot for inter-event intervals showing that octopamine increased the frequency of mEPSCs in a neuron shown in A. The inset is the pooled data showing that frequency of mEPSCs was significantly increased. (*C*) No significant change in the amplitude of mEPSCs after octopamine application in transgenic mice.

The results have indicated that octopamine targeted to presynaptic neurons to increase glutamate release in the ACC in transgenic mice. To further examine the pre- or postsynaptic effect of octopamine, we tested the effects of octopamine on mEPSCs in ACC pyramidal neurons in Ap oa_1 _mice. We found that bath-applied octopamine (50 μM) significantly increased the frequency of mEPSCs from 3.9 ± 0.9 Hz to 5.6 ± 1.1 Hz (n = 8, P < 0.01, paired t-test, Figure 5A and 5B). However, there is no significant change in the amplitude of mEPSCs before and after octopamine application (from 10.5 ± 1.5 pA to 9.6 ± 1.8 pA, n = 8, P = 0.29, Figure 5A and 5C). These results further supported the idea that activation of octopamine receptor by exogenous octopamine increased presynaptic glutamate release in the ACC in Ap oa_1 _mice.

### Microinjection of octopamine into the ACC increases behavioral responses to inflammatory pain

**Figure 6:**
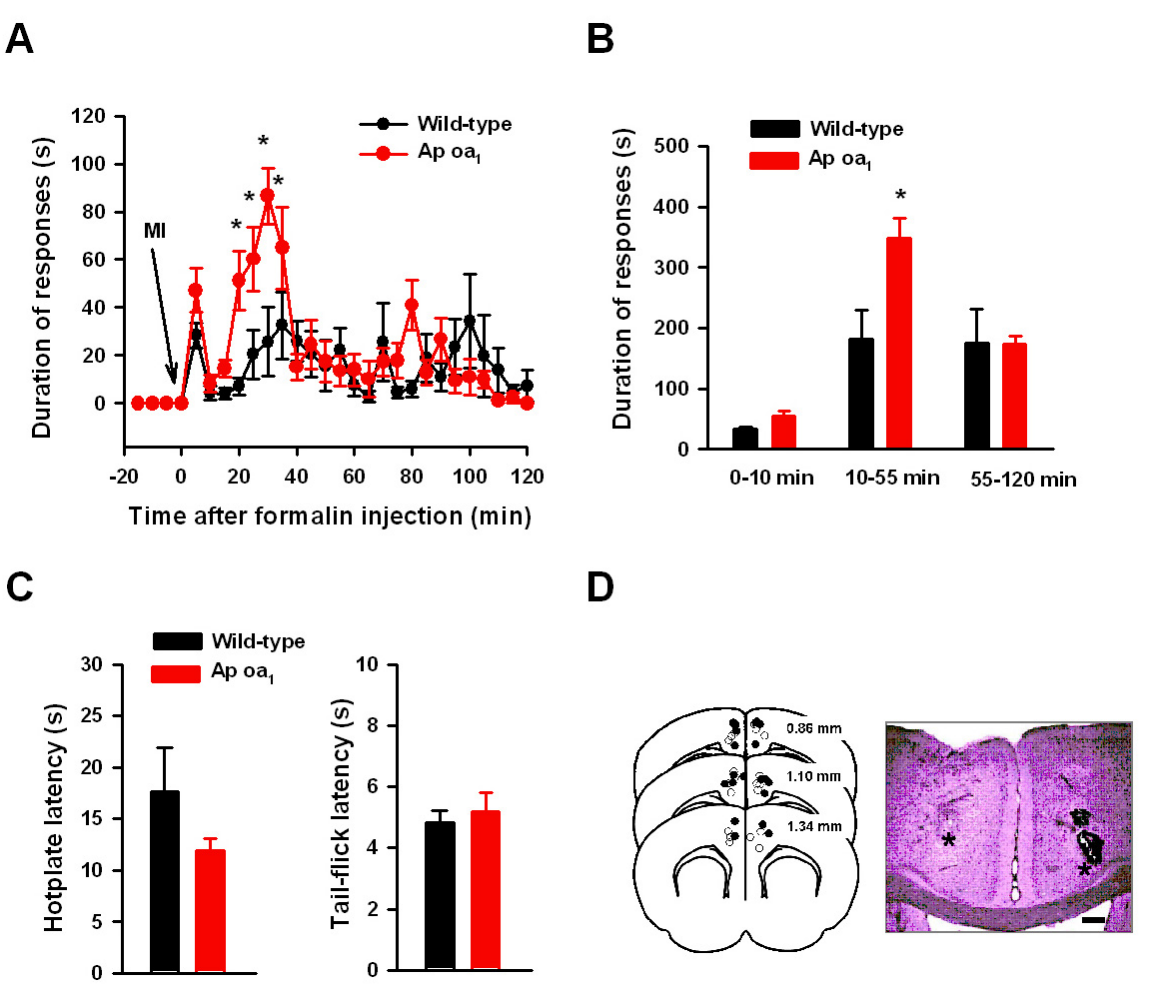
**Microinjection of octopamine at the ACC in Ap oa_1 _mice enhances inflammatory pain**. (*A*) Behavioral nociceptive responses to hindpaw formalin injection, plotted in 5 minute intervals. Wild-type mice are plotted as filled circles (n = 7) while Ap oa_1 _mice are plotted as open circles (n = 6). Arrow shows the time for octopamine microinjection. MI: microinjection. (*B*) Data were grouped into 3 phases: early (acute), intermediate (inflammatory), and late phases. (*C*) Bilateral microinjection of 1 mM octopamine into the ACC of Ap oa_1 _mice (n = 5) did not alter hotplate (left) or tail-flick (right) responses compared to wild-type control (n = 3). (*D*) Location of lesion sites from all animals included in the study (left). Open circles represent injection sites of wild-type mice while filled circles represent injection sites of Ap oa_1 _mice. The right panel showed the example of lesion site location in the ACC of an H&E stained brain section. The injection sites are indicated by *, and the scale bar is 300 μm.

We have previously shown that inflammatory pain caused synaptic alterations in the ACC. For example, presynaptic glutamate release is enhanced while postsynaptic NMDA NR2B subunit is upregulated in the ACC pyramidal neurons after peripheral inflammation [9,10]. However, there is no study on the regional and temporal manipulations in synaptic transmission in the ACC and persistent pain. Since endogenous octopamine is very low [36], we could selectively activate cAMP pathway through local bilateral ACC microinjection of octopamine while testing behavioral response to the inflammation. In a mouse model of inflammatory pain (the formalin test), we found that there was a significant effect of bilateral octopamine (1 mM) injection into the ACC in the formalin test (F_(1,286) _= 5.46, P < 0.037, two-way ANOVA, Figure 6A). Similarly, when data were grouped according to the phase of the formalin test there was a significant effect of octopamine treatment (F_(1,36) _= 5.89, P < 0.032, two-way ANOVA) and the effect occurred exclusively in the intermediate inflammatory phase (Phase 2) (q = 5.36, P < 0.001, Figure 6B). By contrast, the acute phase 1 (0–10 min) (Figure 6A) was apparently unaltered, indicating that the enhancement is selective for late phase responses. This finding is consistent with our previous studies that Phase 2 inflammatory responses were selectively reduced in genetic knockout of AC1 or 8 mice [28]. Finally, we also examined the effects of ACC octopamine microinjection on acute nociceptive responses including the tail-flick and hot-plate tests. We found that octopamine at the ACC did not effect tail-flick (t = 0.38, P = 0.716, Figure 6C), or hot-plate (t = 1.29, P = 0.243, Figure 6C) responses. Taken together, we suggest that the octopamine at the ACC acting on octopamine receptors enhanced inflammatory pain but not acute pain.

## Discussion

**Figure 7:**
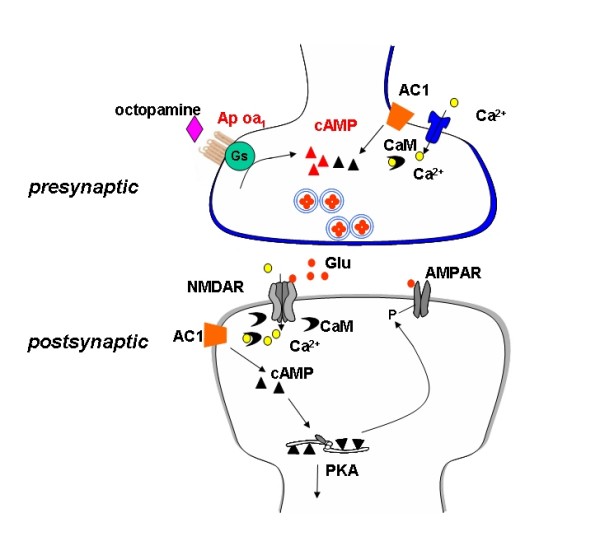
**Proposed model for presynaptic Ap oa_1 _in the synapse in the ACC from transgenic mice**. Diagram showing that activation of presynaptic Ap oa_1 _leads to the production of cAMP. cAMP-related signaling pathways facilitate the glutamate release from presynaptic terminal. cAMP produced by activation of Ap oa_1 _in the presynaptic terminal may mimic the effects of cAMP produced by activation of presynaptic calcium-stimulated AC1 [30]. The released glutamate then acts on postsynaptic glutamate receptors, such as AMPA receptors (AMPAR) and NMDA receptors (NMDAR), and trigger the downstream signaling pathways. In the transgenic mice, Ap oa_1 _is preferentially expressed in the presynaptic terminal in the ACC.

In the present study, we used novel transgenic mice heterologously expressing Ap oa_1 _receptors to examine the role of cAMP pathway in synaptic transmission and chronic pain in the ACC. The use of exogenous receptors is an innovative method that applies the precision of molecular biological techniques to studies on neuronal network. We found that activation of Ap oa_1 _receptors in the ACC enhanced presynaptic glutamate release (Figure 7) as well as behavioral responses to chronic pain. This is the first attempt to directly address the G protein-coupled cAMP pathway in synaptic transmission and its functional significance in the ACC. Our results suggest that increasing presynaptic glutamate release in the ACC is sufficient for enhanced chronic pain.

Since Ap oa_1 _receptor expression is driven by CaMKIIα promoter, the receptor is selectively expressed in forebrain neurons. Therefore, cAMP pathway coupled to Ap oa_1 _receptor could be spatially activated. Although it has been shown the role of cAMP in spinal dorsal horn in chronic pain [39,40], here we demonstrate that elevation of cAMP in the ACC is sufficient to enhance the behavioral sensitization by using the unique mice. In addition, up-regulation of cAMP mainly happens in neurons rather than glial cells, in which cAMP pathway is also activated by AC activator [41,42]. Another advantage for using the transgenic mice is that Ap oa_1 _receptor is *Aplysia *Gs protein-coupled receptor and is thus not activated endogenously. Therefore, exogenous application of octopamine will be the only way of activating Ap oa_1 _receptor and the associated cAMP pathway. Consistently, we have shown here that heterologous expression of Ap oa_1 _receptor does not affect neuronal excitability and basal synaptic transmission. Hence, unlike conventional knockout mice or application of drugs like forskolin [28,29], the Ap oa_1 _transgenic mice provide the unique tool to study functions of G protein-coupled cAMP pathway at cellular and behavioral levels.

### Mechanisms for enhancement of excitatory synaptic transmission by activation of Ap oa_1 _receptor

Electrophysiological experiments have shown that excitatory synaptic transmission in the ACC is primarily glutamatergic and mediated by NMDA, AMPA and kainate receptors [9,43] (Figure 7). Heterologous expression of Ap oa_1 _receptor in mice forebrain is not affecting either NMDA or non-NMDA receptor-mediated synaptic transmission, suggesting no endogenous activation of Ap oa_1 _receptor. However, exogenous application of octopamine, which is known to selectively activate Ap oa_1 _and subsequently increase cAMP level [32,44], significantly enhanced the amplitude of EPSCs. Three lines of evidence showed that the facilitation is due to increased presynaptic release of glutamate. First, paired-pulse ratio is decreased after application of octopamine, indicating the presynaptic component in the modulation. Second, octopamine did not affect the glutamate-induced current in the ACC pyramidal neuron, showing minor postsynaptic regulation on the function of glutamate receptors. Third, the frequency but not amplitude of mEPSCs is increased by bath application of octopamine, consistently suggesting the enhanced presynaptic glutamate release rather than postsynaptic receptor functions. Given that Ap oa_1 _receptor is coupled to Gs protein and elevation of cAMP [32], our results is consistent with the well-known role of cAMP pathway in modulation of neurotransmitter release [14-16]. Taken together, we found that exogenous activation of Ap oa_1 _facilitates excitatory synaptic transmission via increasing presynaptic glutamate release. Thus, these heterologous receptors are probably expressed in presynaptic terminals modulating vesicle release. It will be of interest to determine how Ap oa_1 _receptors traffic and function in these synaptic terminals. However, since the exact anatomic location of the expressed octopamine receptors is unknown, it is important to know whether octopamine has any effect on other membrane conductance and channels (other than glutamate receptors) that would alter postsynaptic excitability.

We found that activation of Ap oa_1 _receptor did not modulate postsynaptic function. The results is surprising, given that cAMP-PKA pathway is known to modulate AMPA receptor phosphorylation and trafficking [18,45]. Currently, we have no clear explanation as to why activation of postsynaptic Ap oa_1 _receptor and following cAMP pathway is not effective in enhanced postsynaptic AMPA receptor function. One possibility is the washout of intracellular molecules by conventional whole-cell patch clamp recordings. This could explain the pre- but not postsynaptic effect by Ap oa_1 _activation in modulating of synaptic transmission. Another possibility is that the cAMP-PKA pathway is important for AMPA trafficking to extrasynaptic but not synaptic sites [46]. If this is the case, activation of Ap oa_1 _receptor may facilitate LTP induction in the ACC. Our previous results have shown that Ca^2+ ^stimulated AC1&8 is critical in cingulate LTP [27], it will be noteworthy to know how elevation of cAMP by G-protein receptor will affect synaptic plasticity in the ACC.

### Functional significance of cAMP pathway and presynaptic glutamate release in the ACC

Our knowledge of the pain pathway and transmission has significantly improved over the last decade; however, the cellular mechanisms of chronic pain remained to be elucidated. Previously, we have shown genetic elimination of behavioral sensitization in AC1&8 knockout mice [28,29]. AC1&8 is coupled to NMDA receptor-induced cytosolic Ca^2+ ^increases to cAMP signaling pathways [19]. Our previous results, therefore, indicate that Ca^2+^-stimualted cAMP pathway is necessary for chronic pain behaviors. In the current study, we extend the aforementioned findings showing that selective elevation of cAMP is sufficient to enhance chronic pain. Most remarkably, using the unique transgenic mice, we provide the first evidence that (1) Selective activation of G protein-coupled receptors and its cAMP pathway in the ACC can enhance behavioral sensitization to pain; and (2) increased presynaptic glutamate release by activation cAMP pathway in the ACC is sufficient for the chronic pain phenotype. In support of the notion that cAMP and glutamate release are critical for chronic pain, our recent study found that enhanced glutamate release in the ACC after chronic pain is also mediated by cAMP pathway [10].

In addition to its role in chronic pain, the cAMP signaling pathway is well-known to be involved in different forms of memory, such as spatial and emotional memory [11,13]. The ACC has diverse functions, including emotion, memory and pain [23,37,47]. Therefore, future experiments will be performed to address the role of the cAMP pathway in ACC-related functions like emotion and memory other than chronic pain using these novel transgenic mice.

## Competing interests

The authors declare that they have no competing interests.

## Authors' contributions

LJW carried out all electrophysiological experiments and drafted the manuscript, HS and SK participated in the behavioral experiments. CI and TA provided transgenic mice. BK and MZ conceived of the study, and participated in its design and coordination. All authors read and approved the final manuscript.
